# Analysis of *S. Epidermidis icaA* and *icaD* genes by polymerase chain reaction and slime production: a case control study

**DOI:** 10.1186/1471-2334-13-242

**Published:** 2013-05-25

**Authors:** Shusheng Zhou, Xiaoguang Chao, Mingming Fei, Yuanyuan Dai, Bao Liu

**Affiliations:** 1 Department of Critical Care Medicine, Affiliated Provincial Hospital of Anhui Medical University, Hefei, China; 2 Department of Laboratory, Affiliated Provincial Hospital of Anhui Medical University, No. 17, Lujiang Road, Hefei, 230001, China

**Keywords:** Catheter-associated blood stream infections, *S. epidermidis*, Slime, *icaA/D*, Scanning electron microscopy

## Abstract

**Background:**

*Staphylococcus epidermidis* is a common pathogen in medical device-associated infections and have an ability to form adherent slime. We aimed to study the effects of *icaA* and *icaD* genes on the slime formation of *Staphylococcus epidermidis* associated with catheter-associated infections.

**Methods:**

*S. epidermidis* isolates from the central venous catheter blood of patients with catheter-associated infections, and from the nasal vestibules of healthy volunteers, intensive care unit hospital staff, and patients, were collected. Slime phenotype was determined by Congo red agar test. The *ica*A/D was detected by polymerase chain reaction. Slime was examined using scanning electron microscopy.

**Results:**

A total of 82 *S. epidermidis* isolates were collected. We found a statistically significant difference with regards to slime production between the clinical isolates from the catheter blood specimens and those from the nasal vestibules (p<0.05). All *S. epidermidis* slime positive strains isolated were *icaA* positive. There was a greater correlation between the presence of both *icaA* and *icaD* and the slime production than the single expression of *icaA* or *icaD* and the presence of slime in all groups. The co-expression of *mecA* and *icaD* was associated with enhanced resistance to antibiotics.

**Conclusion:**

*S. epidermidis* bacteria are significant nosocomial pathogens, and *icaA/D* can clarify the adhesion mechanism in the pathogenesis of infections associated with medical devices. This study result could be useful for the development of rapid diagnosis for slime producing and methicillin resistant *S. epidermidis* strains.

## Background

Coagulase-negative staphylococci (CNS) are the most frequent cause of nosocomial blood stream infections [1], in particular, *Staphylococcus epidermidis*, which has emerged as a major pathogen [2]. *S. epidermidis*, a normal inhabitant of human skin and mucous membranes, is the predominant cause of foreign-body-associated infections [3,4]. In addition, *S. epidermidis* is isolated with increasing frequency as the causative pathogen of nosocomial sepsis, and accounts for approximately 30% of all nosocomial blood stream infections [4]. These infections are often indolent and unresponsive to antimicrobials [5], and frequently result in the removal of the adulterated device. In clinical samples, rates of methicillin resistance in intensive care unit settings have been reported to be 55–77%, or even 86% [6-8].

In an early study, the “slime” production by *S. epidermidis* was found to be significantly associated with clinical infections [9]. Coagulase-negative staphylococci, particularly *S epidermidis*, are the important cause of infections associated with foreign materials. Bacterial adhesion has been considered as the leading cause of severe nosocomial infections related to implanted medical devices [10]. In the recent years, a study has been conducted to investigate the ica gene as a marker of the *S. epidermidis* adhesive aptitude [11].

This study analyzed 82 *S. epidermidis*, collected over a 15-month period from patients, hospital staff in the intensive care unit, and volunteers. The aim of this study was to determine the presence of the *icaA* and *icaD* in *S. epidermidis* from catheter-related blood stream infections and to determine the correlation between the presence of *icaA* and *icaD*, biofilm production, and resistance to antibiotics.

## Methods

### Bacterial strains

This study analyzed 82 *S. epidermidis* isolates collected from May 2009 to July 2010. Twenty-two *S. epidermidis* studied were isolated from patients blood taken from intravascular catheters; another 60 strains of *S. epidermidis* isolated from the nasal vestibules of healthy non-medical volunteers, patients (same patients from whom blood specimens were obtained), and intensive care unites (ICU) hospital staff were also investigated.

All *S. epidermidis* isolates were characterized using vitek 2 compact (bioMe´rieux, France). Ethical approval for the study was obtained from the Central Research Ethics Committee in the Provincial Hospital and included the patients’ and volunteers’ prior written consent.

Two *S. epidermidis* reference strains were used, the well known slime-producing strain ATCC 35984 (RP62A) and the non-slime-producing strain ATCC 12228. The quality control strain was *Staphylococcus aureus* ATCC 25923 [12].

### Strain storage

The *S. epidermidis* were stored in trypticase soy broth (TSB), with 15% glycerol added at -20C for 1 to 15 months long. The isolates were recovered when the study was initiated. The *S. epidermidis* recovery rate reached 100%.

### Study design

This study was a prospective design. Eighty-two strains of *S. epidermidis* were collected over a 15 month period. Eighty-two *S. epidermidis* isolates were collected usng sterile swabs from patients’ central venous catheter blood, and from the nasal vestibules of non-medical volunteers, ICU hospital staff, and patients. A total of 70 volunteers and 69 staff (doctor and nurse) who haven’t had any diseases were included. Among them, the volunteers were 1st year medical students from Anhui Medical University. Only cases with bacteraemia from catheter-associated infections, confirmed by positive blood culture and clinical evidence of catheter-associated infections, were included in the study. Cases without neutropenia and cases with bacteraemia from mixed pathogens were excluded in order to avoid the contamination of *S. aureus* and confusion about the true pathogen. The study was conducted in the ICU of the Affiliated Provincial Hospital, Anhui Medical University, Hefei, China. The unit has the capacity to provide care for 54 patients.

### Detection of slime production

Qualitative detection of the phenotypic production of biofilm formation by all strains was studied by culturing the strains on Congo red agar (CRA) plates (CRA; Sigma Chemical Company, St Louis, MO, USA) [13]. For an accurate assessment of all the possible chromatic variations exhibited by the cultured colonies, a six-color reference scale was used and interpreted, and smooth colonies were classified as slime negative strains.

### Scanning electron micrograph

**Figure 1:**
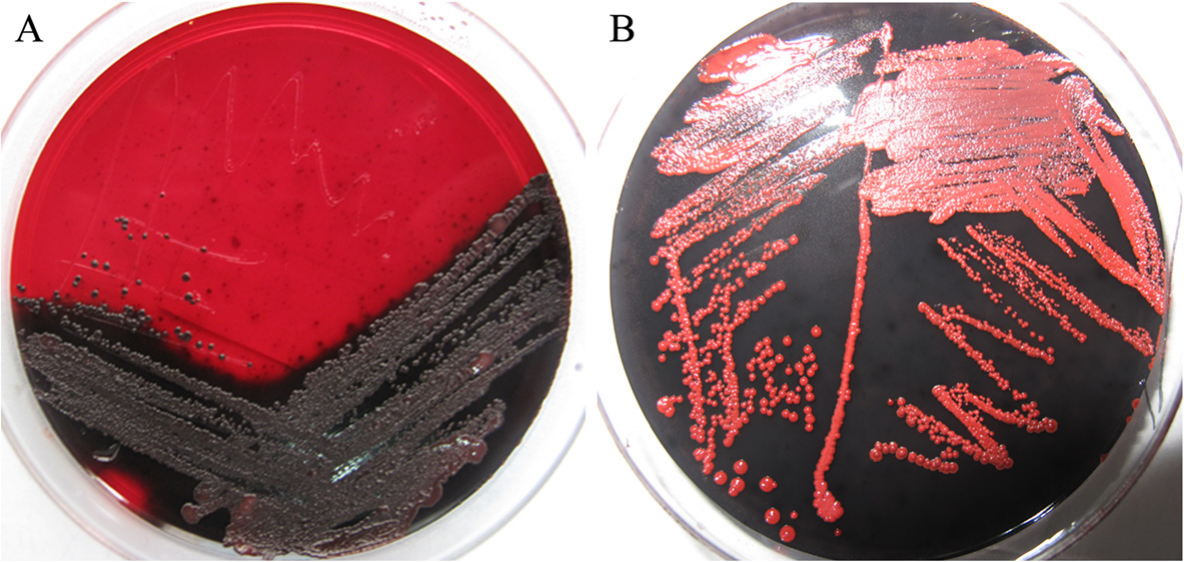
**Glass-surface slime production by cultures on CRA. A**) Black colonies of slime-producing *S. epidermidis* ATCC 35984. **B**) Red colonies of the non-slime-producing *S. epidermidis* ATCC 12228. CRA: Congo Red Agar.

An additional set of experiments was performed using Scanning electron micrograph to evaluate the structure of slime formed under study. Examples of these slimes are shown in Figure 1.

Critical dried material was mounted onto an aluminum stub and coated with palladium in a scanning electron microscopy (JSM-6700 F, JEOL) [14].

### Polymerase chain reaction (PCR) for *icaA and icaD* genes *and mecA* sequences

After overnight culture on brain-heart infusion agar plates, one colony was suspended in 20 ml of sterile distilled water, and the suspension was then heated at 100°C for 20 minutes. From this suspension, a 5 μl aliquot was directly used as a template for PCR amplification.

The sequences of *icaA* and *icaD* were taken from the GenBank sequence database of the National Center for Biotechnology Information [12]. Primers specific for *icaA* and *icaD* were selected based on gene sequences by the Primer 3 program. The primers were synthesized by TAKARA biotechnology (Dalian) Co, Ltd (China).

The presence of *icaA, icaD,* and *mecA* DNA were detected by polymerase chain reaction using forward and reverse primers for *icaA, icaD,* and *mecA*, as described previously [15,16]. For the detection of *icaA* (188-bp), the forward primer had the following sequence: 5′-TCT CTT GCA GGA GCA ATC AA; and 5′-TCA GGC ACT AAC ATC CAG CA was used as a reverse primer. The primer sequences for *icaD* (198-bp) were: forward, 5′-ATG GTC AAG CCC AGA CAG AG; and reverse, 5′-CGT GTT TTC AAC ATT TAA TGC AA. The primers’ detections of the *mecA* (224-bp) were AAA ATC GAT GGT AAA GGT TG GC forward, and AGT TCT GCA GTA CCG GAT TT GC reverse.

PCR was performed in a DNA thermal cycler and using the method described by Petrelli D et al. [17]. The Gene Ruler 100 bp DNA ladder was used as a DNA size marker, and visualized under ultraviolet transillumination.

### Analysis of data

Statistical analysis was performed by means of SPSS 17.0 (SPSS Inc., Chicago, USA) software. Comparisons between groups were performed using the chi-square test. The Pearson correlation coefficient (r) and its significance (P) were calculated between groups; probability values less than 0.05 were considered statistically significant.

## Results

### Slime by *S. epidermidis* isolates

Slime-forming ability of *S. epidermidis* can be inferred by phenotypic effects when grown on Congo red agar. Slime producing strains form rough, black colonies such as those seen in Figure 1A, while the colonies of strains that do not produce slime are red in color, such as those seen in Figure 1B.

### Detection of slime-production

**Figure 2:**
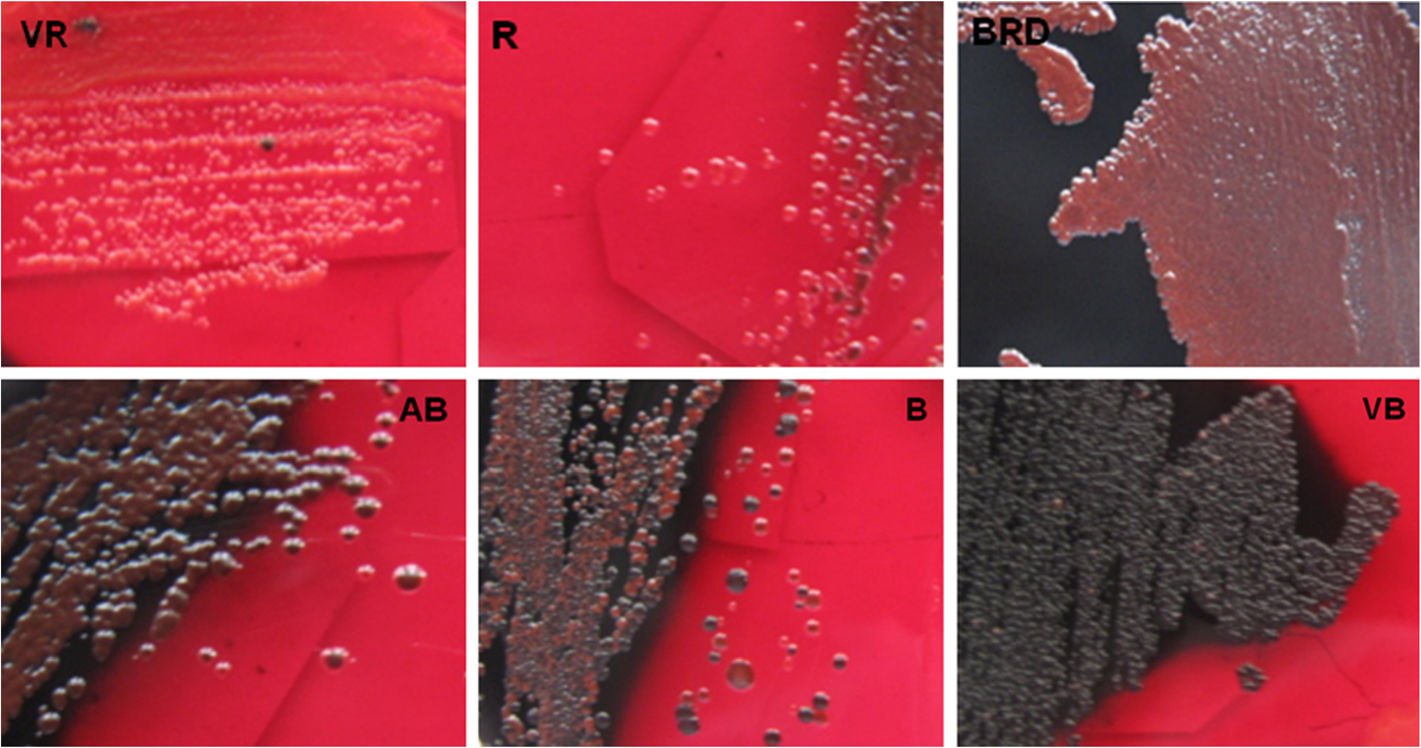
**Colorimetric scale adopted for colony evaluation by Congo red agar. vr**, very red; **r**, red; **brd**, Bordeaux; **ab**, almost black; **b**, black; **vb**, very black.

Sixty five patients who provided the blood isolate were surveyed for nasal carriage and 33.85% (22/65) of them were positive. Phenotypic production of slime by all investigated strains was assessed by culture on CRA plates [13] (Figure 2). Among the clinical isolates from patient catheter blood specimens, 15 of 22 (68.18%) *S. epidermidis* were slime producers and the remaining 7 strains were non-slime producers. The number of slime-producing *S. epidermidis* from the nasal vestibules of healthy non-medical volunteers, patients, and ICU hospital staff were 8 of 21 (38.10%), 5 of 19 (26.32%), and 8 of 20 (40.00%), respectively.

### Scanning electron micrograph of biofilm

**Figure 3:**
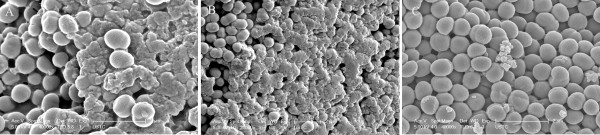
**A scanning electron microscopy image shows a laboratory-grown *Staphylococcus epidermidis *biofilm.** Image **A** shows normal slime-producing strains; **B** shows slime-producing strain ATCC 35984; **C** shows non-slime-producing strain ATCC 12228. Scale bar 2 μm. Image taken by Fu, Instruments’ Center for Physical Science, University of Science & Technology China.

Slime is a biomass of exopolysaccharide that adheres to culture medium surfaces. It appears as clear mucus. (See Figure 3A and B) (Scale bar _2 μm).

### PCR detection of *icaA, icaD,* and *mecA*

**Figure 4:**
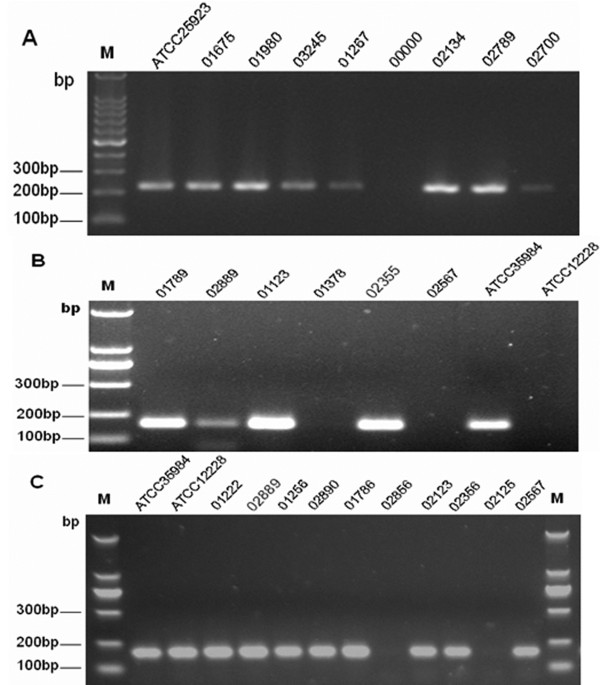
**PCR detection of icaA, icaD, and mecA.** Lane 1, 100 bp DNA molecular size marker. Image A shows *mecA* locus (**A**, 224-bp band), lanes 2–10, PCR amplification of *mecA*, lanes 2, ATCC25923. Lane 7, negative control; lanes 3 to 10, PCR amplicons obtained with DNA of *S. epidermidis*; B shows *icaA* locus (**B**, 198-bp band),lanes 2–9, PCR amplification of *icaA*. Lane 8, ATCC 35984 (RP62A). Lane 9, ATCC 12228. Lanes 2–7, amplification obtained with DNA of clinical *S. epidermidis* isolate. **C** shows *icaD* locus (**C**, 198-bp band), lanes 2–13, PCR amplification of *icaD*. Lane 2, ATCC 35984 (RP62A). Lane 3, ATCC 12228. Lanes 4–13, amplification obtained with DNA of clinical *S. epidermidis* isolate.

The PCR technique was performed on the 82 *S. epidermidis* strains isolated. Typical results are shown in the Figure 4. It was also shown that all strains which were positive for *icaA* were also positive for *icaD*, giving 188-bp and 198-bp band for the *icaA* and *icaD*, and a 224-bp band for the *mecA*. In our study, all isolated slime-positive *S. epidermdis* strains were *icaA* positive, and it was also demonstrated that *icaD* had higher positive rate than *icaA* in all *S. epidermdis* isolates. Also, in this study, a relationship between *mec*A and *icaD* was observed among all four groups (p<0.05). For the clinical isolates from catheter blood, 15 of 22 *S. epidermidis* were found to be positive for both *icaA* and *icaD*, and 15 strains were found to be biofilm producers using the CRA method (Table 1). All 15 slime producing *S. epidermidis* isolates were positive for both icaA and icaD (Table 2).

**Table 1:** **Association between slime production on congo red agar and presence of *icaA *and *icaD *genes in the 82** ***S. epidermidis *strains**

**Groups**	**n**	**Slime production**						***mecA vs icaD***	
		***icaA***		***icaD***		***icaA *+*icaD***			
		**r**	**P value**	**r**	**P value**	**r**	**P value**	**r**	**P value**
1	22	0.894	0.000^◇^	0.459	0.036^△^	1.000	0.000^◇^	0.686	0.001^☆^
2	21	0.679	0.001^◇^	0.439	0.047^△^	0.748	0.000^◇^	0.553	0.009^☆^
3	19	0.701	0.001^◇^	0.406	0.085	0.782	0.000^◇^	0.456	0.049^☆^
4	20	0.533	0.015^△^	0.408	0.074	0.698	0.001^◇^	0.535	0.015^☆^

Group 1, blood isolates obtained from patients’ IV line; Group 2, nasal isolates of healthy volunteers; Group 3, nasal isolates of the same patients in Group 1; Group 4, nasal isolates of ICU staff. ^△^The correlation between the presence of the operon and the presence of slime production in all groups (p<0.05). ^◇^The correlation between the presence of the operon and the presence of slime production in all groups (p<0.001). ^☆^The correlation between the presence of *MecA* and *icaD* (p<0.05).

**Table 2:** **Association between slime production on congo red agar and presence of *icaA *and *icaD *genes in the 82 *S. epidermidis *strains**

**Groups**	**strains (n)**	**Slime Production**	***icaA***	***icaD***	***icaA *+*icaD***	***mecA***
1	22	15 (68.18%)	16 (72.72%)	20 (90.91%)	15 (68.18%)	20
2	21	8 (38.10%)^△^	12 (57.14%)	15 (71.43%)	11 (52.38%)	14
3	19	5 (26.32%)^◇^	8 (42.11%)	15 (78.95%)	7 (36.84%)	14
4	20	8 (40.00%)^◇^	12 (60.00%)	17 (85.00%)	12 (60.00%)	14

Group 1, blood isolates obtained from patients’ IV line; Group 2, nasal isolates of healthy volunteers; Group 3, nasal isolates of the same patients in Group 1; Group 4, nasal isolates of ICU staff. ^△^Values are given as slime production of *S. epidermidis* strains vs first group (p<0.05), ^◇^Values are given as slime production of *S. epidermidis* strains vs first group (p<0.001). There was no statistical difference among other three groups (P>0.05).

## Discussion

*Staphylococcus epidermidis* are found ubiquitously, residing on human skin in healthy adults [18]. It has now been recognized as a key pathogen involved in adhereing to indwelling medical devices surfaces and form biofilms. The major virulence factor is that they can form biofilm on polymeric surfaces and adherence to catheters and other artificial materials during the early phase of biofilm development [13]. Slime production seems, therefore, to be a very important mechanism of adhesion onto biomaterials. In particular, the current study demonstrated that *S. epidermidis* should be recognized as a major cause of catheter infections. This study investigating the biofilm formation showed that slime accumulation is mediated by the chromosomal *ica* gene, which comprises four intercellular adhesion genes (*icaA, icaB, icaC* and *icaD*) and one regulator gene (*icaR*) [19].

### Detection of slime-production

The results of our study are similar to previous literature, in which the percentage of slime-producing strains of *S. epidermidis* ranged from 31% to 89% [20,21]. In addition, we found a statistical difference with regards to slime production between the clinical isolates from the patient catheter blood specimens and isolates from the nasal vestibules of three groups (p<0.05) (Table 2). This indicates that slime is produced in higher proportion in the blood group than in the other groups. The results also indicate that the internal *in vivo* environment can offer a satisfactory biological environment for producing slime.

Our study also found that slime production was reduced in some *S. epidermidis* [43.90% (36/82)]. In this investigation, some strains were found to gradually change from black or bordeaux to red or very red in 3 to 5 generations. We could not find similar results in any literature. Two hypotheses can be proposed: (1) there are many differences in nutrients, pH, oxygen radicals, and antibiotics between *in vivo* and in vitro environmental conditions which may confer a positive selective pressure on slime production; and (2) certain genes such as *icaA* or *icaD* may be lost or mutated. In addition, we conjecture that the virulence factor of *S. epidermidis* will be weak. We believe that a future study of the presence and expression of genes (such as the *icaA* or *icaD* after passage may help clarify the relevance of slime, virulence factor of *S epidermidis*, and *ica*.

### Scanning electron micrograph

In this observation, we intensely studied *S. epidermidis* slime by Scanning electron micrograph. In some studies, the monomeric carbohydrate moieties and several amino sugars are the most distinctive features of a microbial biofilm produced by *S. epidermidis*[22,23]. This is particularly true that slime layer was a suitable intermedia for *S. epidermidis* growth and may cause a subclinical chronic to severe fatal infection [24].

### *icaA* and *icaD* and slime production

Coagulase-negative staphylococci, particularly *S. epidermidis*, are the major causes of infections associated with catheters and other artificial materials. Usually considered to be of low virulence, *S. epidermidis* is now recognized as a potential pathogen because of its ability to produce biofilm and adhere to the walls of artificial materials. In other evidences, the biofilm accumulation is mediated by certain genes, such as *icaA, icaB, icaC, icaD* and *icaR*[25,26]. The recent findings point to an important role of the *icaA and icaD* due to their ability to produce slime strongly in a high percentage of clinical isolates collected from patients with catheters associated infection [10]. Cafiso V reported that the co-expression of *icaA* with *icaD* can increase slime production remarkably [19].

In our investigation, all isolated *S. epidermdis* slime-positive strains were positive for *the icaA*, which is in agreement with other studies [15]. In addition, our investigation demonstrated that *icaD* has a higher positive detection rate. Slime production depends on the presence of both *icaD* and *icaA.* The reason for the absence of biofilm production in some icaA and icaD positive isolates in the present study may be the lack of icaC [27].

In this study we found a stronger correlation between the presence of the *icaA* and *icaD* and the presence of slime production than the single expression of *icaA* or *icaD* and the presence of slime production in all groups (Table 2), and consequently, these results support the idea that the co-expression of *icaA* and *icaD* is associated with enhanced slime production. Our experimental evidence supports the result of Cafiso V [19], and another study demonstrated that co-expression of *icaA* and *icaD* was associated with enhanced slime production [21].

### *MecA* and *icaD* genes

In this study, a relationship was observed between *mec*A and *icaD* among the four groups, and in another investigation of ours, there was a significant association between a positive PCR result for *icaD* and a positive CRA test result for *S. epidermidis*, which indicates the important role of *icaD* as markers in *S. epidermidis* infections. In fact, in recent studies [28,29], *S. epidermidis* is the most common cause of clinical infections because of strains that are able to produce a polysaccharide slime depending on the presence of *ica*, such as *icaD*. This finding suggests the possibility that co-expression of *mecA* and *icaD* is associated with enhanced clinical isolates that produce an extracellular matrix called slime and may make them more resistant to antibiotics. The results of our study are similar to Cafiso V’s study, which suggested that the *ica* and *mecA* could be considered as important virulence markers for clinical *staphylococcal* isolates [19]. Therefore, the demonstration of *mecA* and *icaD* in clinical isolates is useful for the rapid determination of the severity of *S. epidermidis* infection.

Limitations of this study included small numbers of cases, single study site. A future study about the mechanism of the *ica* with prospective design and more numbers of cases will enhance our understanding of the pathogenesis of CVC-related BSI and provide useful information for the development of more effective therapeutic measures to eradicate biofilm in hospitals.

## Conclusion

In summary, these data suggested that *ica* is typically associated with biofilm production of *S. epidermidis* in ICU. Both *icaA* and *icaD* can support the adhesion mechanisms of *S. epidermidis* involved in the infections associated with medical devices. Co-expression of *mecA* and *icaD* is associated with enhanced clinical isolates that produce slime and may be more resistant to antibiotics. This study may help the development of rapid diagnosis approach for slime producing and methicillin resistant strains in hospitals.

## Abbreviations

CNS: Coagulase-negative staphylococci; CRA: Congo red agar; ICU: Intensive care unites; PCR: Polymerase chain reaction; TSB: Trypticase soy broth.

## Competing interests

We declare that we have no dual or conflicting interests.

## Authors’ contributions

SZ participated in the design of the study and performed the statistical analysis. XC participated in the PCR(icaA and icaD genes and MecA sequences) and Scanning electron micrograph. MF cultured the bacteria on CRA plates and assessed the Phenotypic production of slime of all investigated strains. YD carried out the collection and cultivation of bacteria. BL participated in its design and coordination and helped to draft the manuscript. All authors read and approved the final manuscript.

## Pre-publication history

The pre-publication history for this paper can be accessed here:

http://www.biomedcentral.com/1471-2334/13/242/prepub
